# Mediators and moderators of the effect of the game changers for cervical cancer prevention intervention on cervical cancer screening among previously unscreened social network members in Uganda

**DOI:** 10.1186/s12885-023-10924-0

**Published:** 2023-05-11

**Authors:** Joseph KB Matovu, Glenn J. Wagner, Margrethe Juncker, Eve Namisango, Kathryn Bouskill, Sylvia Nakami, Jolly Beyeza-Kashesya, Emmanuel Luyirika, Rhoda K. Wanyenze

**Affiliations:** 1 School of Public Health, Makerere University, Kampala, Uganda; 2 Faculty of Health Sciences, Busitema University, Mbale, Uganda; 3 RAND Corporation, Santa Monica, CA USA; 4 Rays of Hope Hospice Jinja, Jinja, Uganda; 5 African Palliative Care Association, Kampala, Uganda; 6 Mulago Specialized Women and Neonatal Hospital, Kampala, Uganda; 7 School of Medicine, Makerere University, Kampala, Uganda

**Keywords:** Cervical cancer, Prevention, Advocacy, Intervention, Mediation, Moderation

## Abstract

**Introduction:**

Cervical cancer (CC) rates are high in Uganda, yet CC screening rates are very low. Our peer advocacy group intervention, Game Changers for Cervical Cancer Prevention (GC-CCP), was shown to increase CC screening uptake among social network members. In this secondary analysis, we examined mediators and moderators of this effect to better understand how and for whom the intervention was most successful in promoting CC screening.

**Methods:**

We conducted a pilot randomized controlled trial of GC-CCP in Namayingo district, Eastern Uganda between September 2021 and April 2022. Forty adult women who had screened for CC in the past year (index participants) enrolled at baseline: 20 were randomized to receive the 7-session intervention to empower women to engage in CC prevention advocacy, and 20 were assigned to the waitlist control; from these index participants, 103 unscreened social network members (alters) also enrolled. All participants were assessed at baseline and month 6 follow-up. Change in cognitive and behavioral CC-related constructs from baseline to month 6 were examined as mediators, using multivariate linear regression analysis. Index and alter demographics and index CC treatment status were examined as moderators.

**Results:**

Increased alter engagement in CC prevention advocacy fully mediated the intervention effect on alter uptake of CC screening, and was associated with an increased likelihood of alter CC screening. CC treatment status of the index participant was the sole moderator of the intervention effect, as those in the intervention group who had screened positive and received treatment for pre-cancerous lesions were more likely to have alters who got screened for CC by month 6.

**Conclusion:**

The effect of GC-CCP on alter CC screening is greater when the alter reports increased engagement in her own advocacy for CC prevention with others. The intervention effects on increased engagement in CC prevention advocacy among both index and alter participants suggest a diffusion of advocacy, which bodes well for dissemination of knowledge and screening activation throughout a network and the larger community.

## Introduction

The World Health Organization (WHO) estimates that implementation of cost-effective and evidence-based interventions, including human papillomavirus (HPV) vaccination of girls, screening and treatment of precancerous lesions, and improving access to diagnosis and treatment of invasive cancers will reduce the median cervical cancer (CC) incidence rate by 97% by 2120– averting more than 74 million new cases of cervical cancer between 2020 and 2120 [1, 2]. A recent comparative modeling analysis of two CC prevention interventions (HPV vaccination and CC screening) in 78 low to middle income countries concluded that HPV vaccination coverage of up to 90% can lead to CC elimination in most countries. However, this is not sufficient to reduce the burden of CC in countries with the highest CC incidence (> 25 cases per 100 000 women-years) [3]. Brisson et al. [3] argue that introducing twice-lifetime screening, in addition to HPV vaccination, will accelerate elimination by 11–31 years and will be necessary to eliminate CC in countries with the highest incidence. These findings highlight the crucial need for innovative interventions to expand access to CC screening services particularly in sub-Saharan Africa which holds 90% of the CC burden.

**Fig. 1:**
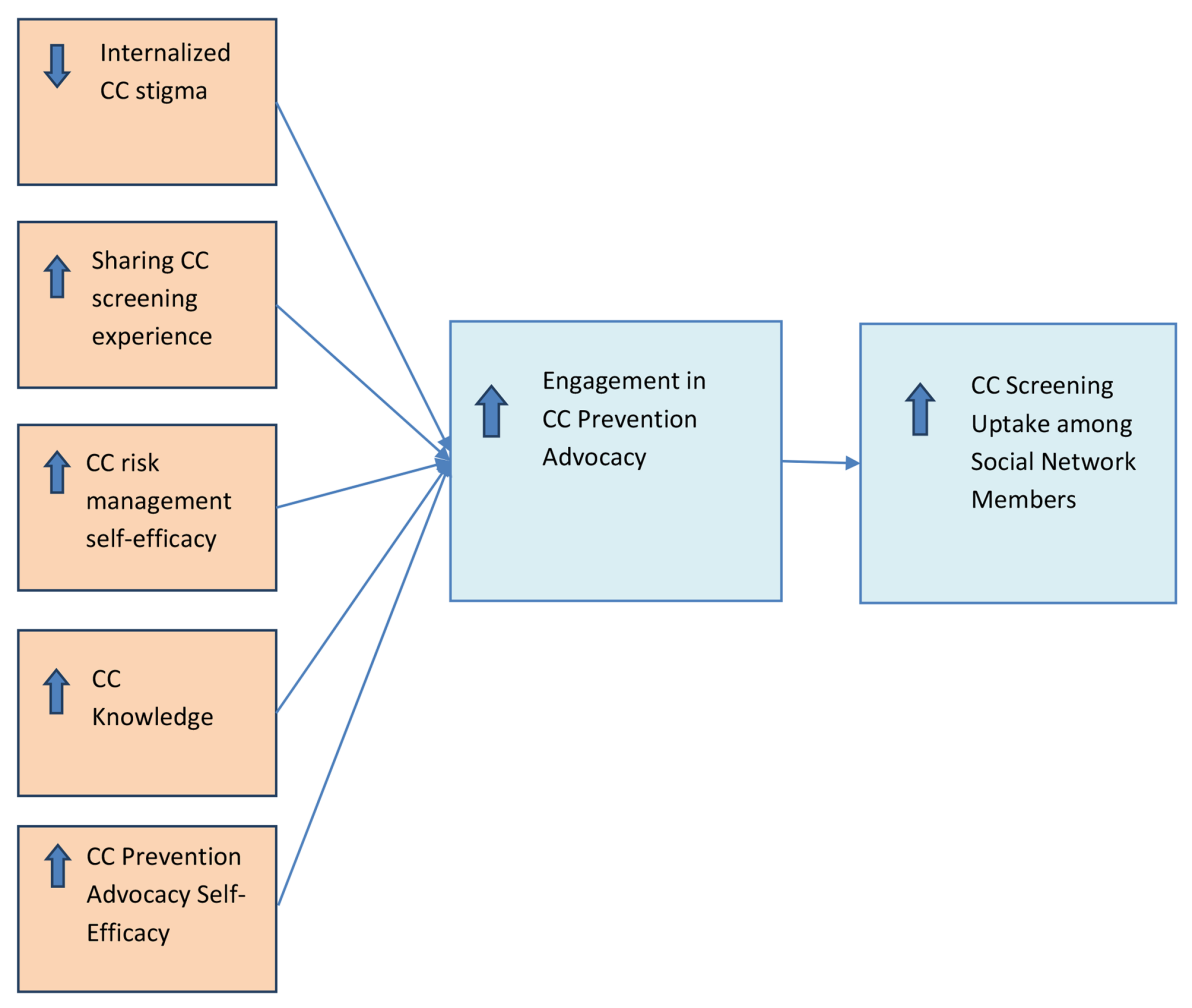
Conceptual framework for promotion of cervical cancer (CC) prevention advocacy among screened women to affect CC screening among social network members

Several interventions have been implemented to increase early detection of CC among women globally, including health education interventions [4, 5]; economic incentivization interventions [6]; and innovative service delivery models such as HPV self-sampling and integration of cervical screening with other services to make cervical screening more comfortable, convenient, and accessible [7]. Despite the availability of these interventions, uptake of CC screening among eligible women remains low in much of sub-Saharan Africa [8, 9]. Building on theories of social diffusion [10], cognitive consistency [11] and social influence [12], which posit that behavior change can be initiated by a few and diffused to others through modeling, advocacy, and shifts in social norms, we developed a cervical cancer prevention intervention, Game Changers for Cervical Cancer Prevention (GC-CCP). As depicted in Fig. 1, this peer-facilitated group intervention seeks to empower and mobilize women who have recently been screened for CC to act as change agents for CC screening within their social networks by directly targeting stigma reduction, sharing of CC screening experience, knowledge of CC facts and myths, CC risk management, and advocacy skills building. Training women who have screened to act as advocates for early CC screening by sharing their personal experience with screening, providing accurate information about CC disease, screening and treatment, and encouraging early screening can help to alleviate the fears and address misconceptions that impede women from getting screened for CC.

We conducted a pilot randomized controlled trial of GC-CCP and found that the intervention led to increased engagement in CC prevention advocacy, and uptake of CC screening in over half of the previously unscreened women in the social networks of the intervention group, compared to less than one-fifth of their counterparts in the control group (Wagner et al., *under review*), the latter of which is similar to the national estimate of lifetime CC screening in Uganda [13, 14]. To understand how the intervention affects screening uptake, and for whom the intervention was most effective, we conducted secondary analyses to examine mediators and moderators of the intervention effect on CC screening uptake among previously unscreened social network members. With increased engagement in CC prevention advocacy being the most direct target of the intervention, our primary hypothesis was that intervention recipient’s engagement in CC prevention advocacy would mediate the intervention’s effect on increased CC screening among social network members.

## Methods

### Study setting

This analysis uses data collected as part of a randomized control trial of GC-CCP in Eastern Uganda between September 2021 and April 2022. The pilot study was conducted at Buyinja Health Center IV and Banda Health Center III in Namayingo, a rural community in the Busoga region of Uganda. The selection of the study site was influenced by previous work in this area by our partner institution (Rays of Hope Hospice Jinja; RHHJ) coupled with low CC screening rates observed among women in this district. CC screening with visual inspection (VIA) and thermal therapy are available at both Buyinja and Banda health centers, and from RHHJ which conducts periodic mobile CC screening and thermal therapy “day camps”. Women who need biopsies are sent to Jinja (approximately 90 km from Namayingo), and if cancerous lesions are present, they are referred to the Uganda Cancer Institute, the leading tertiary public cancer care centre located in Kampala.

### Study design

The study design has been described in detail in our previous publication [15]. In brief, women who had recently screened for CC (referred to as index participants) were randomized to receive the GC-CCP intervention (intervention arm) or assigned to the wait-list control group, with individual randomization on a 1:1 ratio. Women randomized to the wait-list control group received the intervention after data collection was completed. Randomization was stratified by age (under and over age 35) and history of treatment for dysplasia. Participants were not blinded to assignment; only the data analyst was blinded. Each index participant was asked to enroll up to three women in their social network (referred to here as “alters”) who had not screened for CC in the past 3 years (though all alters reported never having been screened). All participants (index and alter) were administered assessments at baseline and month 6, and received 30,000 Uganda shillings (~$8 USD) for each completed assessment. The primary outcome was alter CC screening over the 6-month follow-up period. The trial was registered on ClinicalTrials.gov (NCT04960748) on 14/07/2021.

### Participants

Index participants were enrolled into the study if they were aged 18 years or older, had screened for CC within the past year, had stable health status (i.e., not in end stages of disease, so that they were likely to complete the 6-month study follow-up), and had shared their CC screening experience with at least one woman (alter) who they perceived to not have screened for CC in the past 3 years. Alter participants were eligible if they were at least 18 years of age, were recruited by a woman who was enrolled as an index participant, and self-reported not being screened for CC in the past 3 years. All participants had to speak either Samia or Lusoga, the two prominent languages in Namayingo district.

Recruitment of index participants was purposive in order to recruit a balance of women who screened positive for signs of CC risk (pre-cancerous or cancerous lesions), and women who screened negative, so that we could assess whether this factor was associated with the outcomes measure of engagement in CC prevention advocacy. Candidates for index participation were informed of the study by health care providers and those who expressed interest were referred to the study coordinator for confirmation of eligibility and consent procedures. Women who decided to enroll were administered the baseline survey, which included listing up to 12 women in their social network. To recruit alters, we randomly selected five alters who the index participant reported as knowing her CC screening experience (or as many as there were if less than five); the index participant was then asked if she was comfortable asking three of these alters to participate. The index participant was asked to call each selected alter at the end of the interview to describe the study in the presence of the coordinator, who then scheduled a study visit if the alter expressed interest in participating. If an alter refused or could not be reached, a replacement was randomly selected from the list of alters who knew the index participant’s CC screening experience (and whom the index participant was comfortable recruiting). When screening the alter, the coordinator confirmed that the individual was not already recruited by another index. All participants provided written informed consent.

### Intervention

The intervention has been described previously [15]. In brief, the intervention consisted of seven weekly group sessions. ***Session 1*** focused on addressing fears and concerns related to CC risk and use of self-compassion and peer support to overcome fears and internalized stigma, as well as introducing the overall vision for empowering women to become change agents for CC prevention and treatment. ***Session 2*** focused on building skills and decision making for sharing one’s personal CC screening experience, knowing to whom to disclose and when, and how to initiate and navigate disclosure and conversations about CC. ***Session 3*** built skills and motivation for recognizing signs of CC risk and seeking health services, so that the advocate’s own behavior was consistent with the behavior they encouraged in others, as well as instruction on facts and myths related to CC to facilitate accurate CC screening advocacy. ***Session 4*** introduced the concept of a social network and how one’s network can serve as a tool for CC prevention advocacy and dissemination of CC-related information. ***Sessions 5 and 6*** focused on the skills needed for successful CC prevention advocacy, including strategies for how to start and sustain conversations about CC, and effective communication skills (e.g., reflective listening, paraphrasing, open-ended questions). ***Session 7*** focused on peer solidarity and support to inspire a commitment to ongoing CC advocacy. The sessions were administered in a group format to facilitate the use of: sharing of experiences to build support, solidarity and motivation among participants; group problem solving and role playing to build skills and self-efficacy; setting personal goals regarding disclosure and advocacy; and take home activities to reinforce practice of new skills and generate personal experiences to be processed in the sessions. Each session lasted 120–150 min.

The sessions were conducted using a structured facilitator manual, in the predominant local languages of Samia and Lusoga, by two peer facilitators from Namayingo who themselves had been screened for CC. The facilitators were trained by the senior investigators over three days. The supervisor of the facilitators observed the implementation of each session and provided feedback and further training as needed during weekly supervision.

### Measures

Assessments included a standard survey (index and alter participants) and social network assessment (index participant only), which were administered in either Samia or Lusoga (depending on the preference of the participant) using Network Canvas computer-assisted software. Each measure was assessed with both index and alter participants, unless otherwise noted. Measures were translated using standard translation/backtranslation methodology. CC screening and treatment utilization were verified with abstracted medical chart data. All measures were developed by the study team, except those in which an attribution is cited. For measures developed by the study team that included at least three items, we cite internal reliability statistics (Cronbach’s alpha).

#### CC screening and treatment

Data were collected to determine if the participant had ever been screened for CC using visual inspection using acetic acid (VIA) or pap smear, and if so, when. For participants who had been screened, it was determined if the screening resulted in pre-cancerous lesions or potential cancerous lesions, in separate items; if either type of lesion was reported, receipt of corresponding procedure or treatment (cryotherapy or thermal therapy for pre-cancerous; biopsy to confirm diagnosis, and radiation, chemotherapy or surgery treatment for cancerous) was assessed.

#### Potential mediators

The following measures were assessed as potential mediators of the effects of intervention effect on alter CC screening, as each was targeted by the components of the intervention. *Internalized CC stigma* was assessed among index participants, using 5 items adapted from a scale of HIV internalized stigma (e.g., “My cervical cancer screening makes me feel ashamed of myself” [16]); higher mean score reflects greater stigma. *Sharing of CC screening experience* was assessed by asking respondents to what extent they had shared their CC screening result with sexual partners, family, and friends, in separate questions; higher mean item score reflects greater disclosure (Cronbach’s alpha = 0.74). *CC knowledge* was assessed with 16 statements or questions reflecting the etiology, prevention and treatment of CC; a sum of correct responses was calculated. Internal reliability was moderate (Cronbach’s alpha = 0.75). *CC enacted stigma* was assessed among alter participants with six items adapted from measures developed by Marlow & Wardle [17] and Cho et al. [18]. Participants were asked to rate their agreement with statements (e.g., A woman with cervical cancer is to blame for her condition; I feel uncomfortable when I am around women with cervical cancer) by indicating they 1 ‘disagree’, 2 ‘I neither agree nor disagree. I do not have a feeling either way’ or 3 ‘agree’; mean item score was calculated and higher scores reflect greater stigma. *CC risk management self-efficacy* was assessed with three items that measured confidence to notice a symptom of CC risk, seek health services for a symptom of CC risk, and obtain treatment if screening revealed signs of CC risk; higher mean item score reflects greater self-efficacy. Internal reliability was low (Cronbach’s alpha = 0.64). *CC prevention advocacy self-efficacy* was assessed with three items assessing confidence to start a conversation about the need for: CC screening, treatment for signs of CC risk, and telling someone about their CC screening experience; higher mean item score reflects greater self-efficacy (Cronbach’s alpha = 0.85). *CC prevention advocacy* was assessed with six items in which respondents with the reported frequency of discussing CC-related topics (e.g., importance of CC screening, how and where to get screened, importance of getting treatment if signs of CC risk are present) with women they know in the past six months. Response options ranged from 1 ‘not at all’ to 5 ‘very much’; mean item scores were calculated and higher scores reflect greater engagement in advocacy. Internal reliability was high (Cronbach’s alpha = 0.95*).*

#### Potential moderators

The variables we examined as potential moderators among both index and alter participants consisted of age and any secondary education, in addition to CC-related treatment history (index participants only) and presence of a main sex partner (alter participants only).

### Data analysis

Descriptive and bivariate (2-tailed independent t-tests; chi-square or Fisher’s Exact tests) statistics were used to compare baseline sample characteristics of index and alter participants in the control versus intervention arms, in separate analyses. To examine intervention effects on index and alter measures of CC-related processes, we conducted multiple linear regression analyses. In each model, the month 6 measure of the outcome was the dependent variable, while independent variables included the baseline measure of the dependent variable and an indicator of study arm. If a measure was missing at month 6, the baseline measure of the variable was used to replace the missing value. Normal probability plots were constructed for each dependent variable and examined qualitatively for violation of normality. Similar multiple logistic regression models were run to examine whether alter uptake of CC screening by month 6 was associated with the index and alter measures of CC-related processes.

Measures of index and alter CC-related processes (potential mediators) were examined as mediators of the intervention effect on alter uptake of CC screening if the measure was significantly associated with both the intervention and alter CC screening. To test for mediation, we employed Proc Causalmed (SAS v9.4), which uses bootstrap resampling to compute standard errors and confidence intervals for causal mediation effects and decompositions. A two-step approach was used to test each potential mediator separately. In step one, the dependent variable was alter uptake of CC screening by month 6, while independent variables consisted of an indicator of study arm and the baseline measure of the mediator. In step two, the month 6 measure of the mediator was added to the model, resulting in the model assessing the mediating effect of change in the mediator from baseline to month 6. Covariates included in each model consisted of age < 35 years, any secondary education, and presence of a main sex partner. We examined each potential mediator separately, because the mediators are conceptually inter-correlated, and testing multiple mediators simultaneously would complicate the interpretation of the models. To test for moderation of the intervention effect on alter CC screening, for each potential moderator we ran a logistic regression modeling alter CC screening at month 6 as the dependent variable. Independent variables included an indicator for study arm, the moderator measured at baseline, and their interaction.

### Ethical considerations

The study protocol was reviewed and approved by the Makerere University School of Public Health Research and Ethics Committee, and the Uganda National Council for Science and Technology.

## Results

### Sample characteristics

Of the 40 index participants, 57.5% were older than 35 years, 22.5% had any secondary education, 87.5% reported that they had a main sexual partner, 92.5% had children, and 7.5% were HIV-positive. There were no significant differences between the index participants randomized to the intervention and those that were randomized to the control arm. Among the 103 alter participants, 37.9% were older than 35 years of age, 29.1% had any secondary education, 81.6% reported that they had a main sexual partner, 95.1% had children, and 5.8% were HIV-positive. Compared to alters of index participants in the control arm, alters of index participants in the intervention arm were significantly more likely to have any secondary education (39.7% vs. 15.6%; p = 0.01) and to report that they had a main sex partner (89.7% vs. 71.1%; p = 0.02).

### Mediation of the intervention effect on alter CC screening by index participant measures

Table 1 (lower panel) shows the associations between alter uptake of CC screening at month 6, and index measures of CC-related processes at month 6, after controlling for the baseline measure of the mediators. Alters who screened for CC were recruited into the study by index participants who reported greater sharing of their CC screening result with others, higher CC knowledge, and greater engagement in CC prevention advocacy and CC screening advocacy across all their named alters, compared to index participants who recruited alters that did not screen for CC by month 6.

**Table 1:** Bivariate and multivariate correlates of cervical cancer (CC) screening among alters

Variable	Not screened (n = 59)	Screened (n = 44)	P value	OR (95% CI)*
**Index CC-related processes at month 6**				
CC internalized stigma	**1.07 (0.23)**	**1.00 (0)**	**0.02**	0 (0, -); p = 1.0
Sharing CC screening experience	**1.65 (0.48)**	**1.91 (0.23)**	**< 0.001**	**12.94 (2.99, 56.07)**
CC knowledge	**11.34 (3.69)**	**14.45 (3.32)**	**< 0.001**	**1.28 (1.12, 1.47)**
CC risk management self-efficacy	8.77 (1.87)	9.30 (1.77)	0.15	1.15 (0.91, 1.45)
CC prevention advocacy self-efficacy	9.26 (1.49)	9.51 (1.32)	0.19	1.08 (0.80, 1.46)
CC prevention advocacy	**3.90 (1.20)**	**4.59 (0.94)**	**0.001**	**2.03 (1.29, 3.19)**
Mean CC screening advocacy across all alters	**2.06 (0.11)**	**2.19 (0.22)**	**< 0.001**	**105.8 (6.4, 1735.2)**
**Alter CC-related processes at month 6**				
CC knowledge	**7.36 (3.80)**	**11.55 (3.62)**	**< 0.001**	**1.31 (1.15, 1.48)**
CC prevention advocacy	**1.88 (0.86)**	**3.70 (1.32)**	**< 0.001**	**3.43 (2.19, 5.37)**
CC risk management self-efficacy	**7.15 (2.04)**	**9.63 (4.85)**	**< 0.001**	**1.87 (1.31, 2.66)**
CC screening self-efficacy	1.99 (0.04)	1.99 (0.03)	0.48	513 (0.00, 73 mil)
CC enacted stigma	1.66 (0.42)	1.58 (0.35)	0.29	0.52 (0.18, 1.56)

* Bivariate analysis was conducted using 2-tailed independent t-tests. Multiple logistic regression models of alter uptake of CC screening included the month 6 measure of alter CC screening as the dependent variable, and the month 6 and baseline measures of the predictor as independent variables

OR = odds ration; CI = confidence interval

Table 2 shows the results from linear regression models of the intervention effects on index measures at month 6, after controlling for the baseline measure of the mediator. Index participants in the intervention arm reported more sharing of their CC screening results with others, and higher levels of CC knowledge, CC risk management self-efficacy, CC prevention advocacy self-efficacy, engagement in CC prevention advocacy, and CC screening advocacy across alters, compared to those in the control arm.

**Table 2:** Logistic regression models examining index measures at month-6 as mediators of intervention effect on alter cervical cancer (CC) screening

	CC Knowledge		Sharing CC screening experience		CC prevention advocacy		Mean CC screening advocacy across all alters	
	Without mediator	With mediator	Without mediator	With mediator	Without mediator	With mediator	Without mediator	With mediator
	OR (95% CI)	OR (95% CI)	OR (95% CI)	OR (95% CI)	OR (95% CI)	OR (95% CI)	OR (95% CI)	OR (95% CI)
Intervention	**1.75** **(1.60, 1.91)**	3.49 (0.21, 57.29)	**1.15** **(1.06, 1.24)**	3.72 (0.13, 109.20)	**1.47** **(1.37, 1.59)**	7.78 (0.71, 85.32)	**1.10** **(1.07, 1.12)**	*3.58* *(0.99, 12.90)*
Direct effect		67.15 (0.21, 21868.35)		**7.50** **(1.50, 37.42)**		**70.89** **(4.54, 1106.88)**		**6.95** **(1.72, 28.04)**
CC knowledge (bsln)	**1.01** **(1.00, 1.02)**	1.07 (0.96, 1.19)	--	--	--	--	--	--
CC knowledge (M6)	--	1.06 (0.63, 1.79)	--	**--**	**--**	**--**	**--**	**--**
Sharing CC screening experience (bsln)	--	--	**1.12** **(1.01, 1.25)**	0.62 (0.30, 1.29)	--	--	--	--
Sharing CC screening experience (M6)	--	--	--	1.84 (0.15, 23.06)	--	--	--	--
CC prevention advocacy (bsln)	--	--	--	--	**1.07** **(1.04, 1.10)**	1.09 (0.87, 1.37)	--	--
CC prevention advocacy (M6)	--	--	--	--	--	0.70 (0.26, 1.84)	--	--
Mean CC screening advocacy (bsln)	--	--	--	--	--	--	0.98 (0.92, 1.04)	1.02 (0.47, 2.18)
Mean CC screening advocacy (M6)	--	--	--	--	--	--	--	**4.19** **(1.58, 11.15)**
Indirect effect		0.14 (0.00, 41.10)		*1.48 (0.94, 2.33)*		0.13 (0.01, 1.84)		1.53 (0.80, 2.92)

A two-step approach was used to test each potential mediator separately. In step one (“without mediator column”), the dependent variable was alter uptake of CC screening by month 6, while independent variables consisted of an indicator of study arm and the baseline measure of the mediator. In step two (“with mediator column”), the month 6 measure of the mediator was added to the model. Covariates included in each model consisted of age < 35 years, any secondary education, and presence of a main sex partner

OR = odds ratio; CI = confidence interval; bsln = baseline; M6 = month-6 follow-up visit

With index participant measures of CC knowledge, sharing of CC screening experience, engagement in CC prevention advocacy, and CC screening advocacy across all named alters each being associated with both the intervention and alter CC screening, we then conducted mediation analysis to assess whether each mediated the intervention effect on alter CC screening, in separate logistic regression models. None of the variables were found to mediate the intervention effect (see Table 3).

**Table 3:** Linear regression models of intervention effects on alter cervical cancer (CC)-related processes (mediators) at month 6, controlling for baseline measure of the mediator

Outcome	Baseline			Month 6			
	Control (n = 45)	Intervention (n = 58)	p*	Control (n = 41)	Intervention (n = 57)	p*	Beta (SE); p
CC knowledge	4.76 (2.60)	5.57 (2.83)	0.14	**6.13 (2.93)**	**11.48 (3.60)**	**< 0.001**	**4.80 (0.67); <0.001**
CC enacted stigma	1.76 (0.57)	1.81 (0.58)	0.63	1.60 (0.36)	1.65 (0.41)	0.55	0.07 (0.08); 0.37
CC risk management self-efficacy	6.96 (1.57)	7.40 (1.46)	0.14	**6.59 (2.11)**	**9.47 (4.20)**	**< 0.001**	**2.75 (0.73); <0.001**
CC screening self-efficacy	1.92 (0.12)	1.91 (0.14)	0.36	**1.98 (0.06)**	**2.00 (0)**	**0.03**	0.05 (0.03); 0.10
CC prevention advocacy	1.76 (0.48)	1.83 (0.68)	0.55	**1.69 (0.69)**	**3.41 (1.36)**	**< 0.001**	**1.75 (0.23); <0.001**

* p values from bivariate comparisons using independent 2-tailed t-tests

Beta coefficients and standard errors (SE) are from multiple linear regression models of alter CC-related at month 6 as the dependent variable, and independent variables consisting of intervention condition and the baseline measure of the CC-related process

### Mediation of the intervention effect on alter CC screening by alter participant measures

The first step to identifying potential mediators of the intervention effect on alter CC screening was to identify variables that were associated with the intervention as well as alter CC screening. Starting with alter participant measures, Table 4 shows the results from linear regression models of the intervention effects on alter CC-related processes (mediators) at month-6, after controlling for the baseline measure of the mediator. Alters of index participants in the intervention group were more likely to have higher CC knowledge, higher CC risk management self-efficacy, and greater engagement in CC prevention advocacy compared to alters of index participants in the control group.

**Table 4:** Linear regression models of intervention effects on index cervical cancer (CC)-related processes (mediators) at month 6, controlling for baseline measure of the mediator

Outcome	Baseline			Month 6			
	Control (n = 20)	Intervention (n = 20)	p*	Control (n = 19)	Intervention (n = 20)	p*	Beta (SE); p
Sharing of CC screening result with others	1.47 (0.62)	1.62 (0.39)	0.37	**1.58 (0.48)**	**1.93 (0.21)**	**0.006**	**0.31 (0.11); 0.008**
CC knowledge	8.55 (2.80)	10.05 (3.36)	0.13	**8.50 (2.37)**	**15.70 (0.66)**	**< 0.001**	**6.90 (0.54); <0.001**
CC internalized stigma	1.21 (0.35)	1.07 (0.16)	0.12	1.08 (0.29)	1.00 (0.00)	0.13	-0.04 (0.05); 0.42
CC risk manangement self-efficacy	8.70 (1.40)	8.85 (1.28)	0.73	**7.40 (2.16)**	**10.00 (0.00)**	**< 0.001**	**2.56 (0.47); <0.001**
CC prevention advocacy self-efficacy	9.75 (0.46)	9.72 (0.49)	0.83	**8.15 (1.98)**	**10.00 (0.00)**	**< 0.001**	**1.85 (0.45); <0.001**
CC prevention advocacy	3.23 (1.13)	3.57 (1.16)	0.35	**2.90 (1.10)**	**4.98 (0.11)**	**< 0.001**	**1.94 (0.20); <0.001**
Mean CC screening advocacy across alters	1.96 (0.19)	1.85 (0.51)	0.39	**2.03 (0.06)**	**2.19 (0.21)**	**0.004**	**0.16 (0.05); 0.003**

* p values from bivariate comparisons using independent 2-tailed t-tests

Beta coefficients and standard errors (SE) are from multiple linear regression models of index CC-related at month 6 as the dependent variable, and independent variables consisting of intervention condition and the baseline measure of the CC-related process

Table 1 (lower panel) shows the associations between alter uptake of CC screening at month 6 and alter measures of potential mediators at month 6, after controlling for the baseline measure of the mediator. Alters who had screened for CC had higher CC knowledge, greater engagement in CC prevention advocacy, and higher CC risk management self-efficacy, compared to alters who had not been screened for CC by month 6.

With alter measures of CC knowledge, CC risk management self-efficacy, and engagement in CC prevention advocacy each being associated with both the intervention and alter CC screening, we then conducted mediation analysis to assess whether each measure mediated the intervention effect on alter CC screening, in separate logistic regression models. Change in alter engagement in CC prevention advocacy from baseline to month 6 fully mediated the intervention effect on alter CC screening (see Table 5), as indicated by the significant indirect effect [adj. OR (95% CI) = 5.75 (1.90, 17.40)] and the nonsignificant direct effect [adj. OR (95% CI) = 1.82 (0.50, 6.66)]. The other two variables had no mediation effect.

**Table 5:** Logistic regression models examining alter measures at month-6 as mediators of intervention effect on alter cervical cancer (CC) screening

	CC Knowledge		CC risk management self-efficacy		CC prevention advocacy	
	Without mediator	With mediator	Without mediator	With mediator	Without mediator	With mediator
	OR (95% CI)	OR (95% CI)	OR (95% CI)	OR (95% CI)	OR (95% CI)	OR (95% CI)
Intervention	**1.76 (1.50, 2.08)**	2.53 (0.83, 7.69)	**1.42 (1.18, 1.71)**	**3.29 (1.09, 9.91)**	**2.09 (1.73, 2.51)**	2.07 (0.59, 7.31)
Direct effect	**--**	*4.48 (0.94, 21.20)*	--	**5.97 (1.43, 24.90)**	--	1.82 (0.50, 6.66)
CC knowledge (bsln)	**1.04 (1.01, 1.06)**	1.06 (0.98, 1.14)	--	--	--	--
CC knowledge (M6	--	*1.08 (0.99, 1.17)*	--	**--**	**--**	**--**
CC risk management self-efficacy (bsln)	--	--	**1.04 (1.00, 1.09)**	*1.14 (0.98, 1.32)*	--	--
CC risk management self-efficacy (M6)	--	--	--	1.01 (0.73, 1.40)	--	--
CC prevention advocacy (bsln)	--	--	--	--	**1.24 (1.10, 1.40)**	0.91 (0.69, 1.20)
CC prevention advocacy (M6)	--	--	--	--	--	**1.42 (1.15, 1.75)**
Indirect effect		*2.53 (0.93, 6.93)*		*2.22 (0.90, 5.52)*		**5.75** **(1.90, 17.40)**

A two-step approach was used to test each potential mediator separately. In step one (“without mediator column”), the dependent variable was alter uptake of CC screening by month 6, while independent variables consisted of an indicator of study arm and the baseline measure of the mediator. In step two (“with mediator column”), the month 6 measure of the mediator was added to the model. Covariates included in each model consisted of age < 35 years, any secondary education, and presence of a main sex partner

OR = odds ratio; CI = confidence interval; bsln = baseline; M6 = month-6 follow-up visit

### Moderators of the intervention effect on alter CC screening

**Fig. 2:**
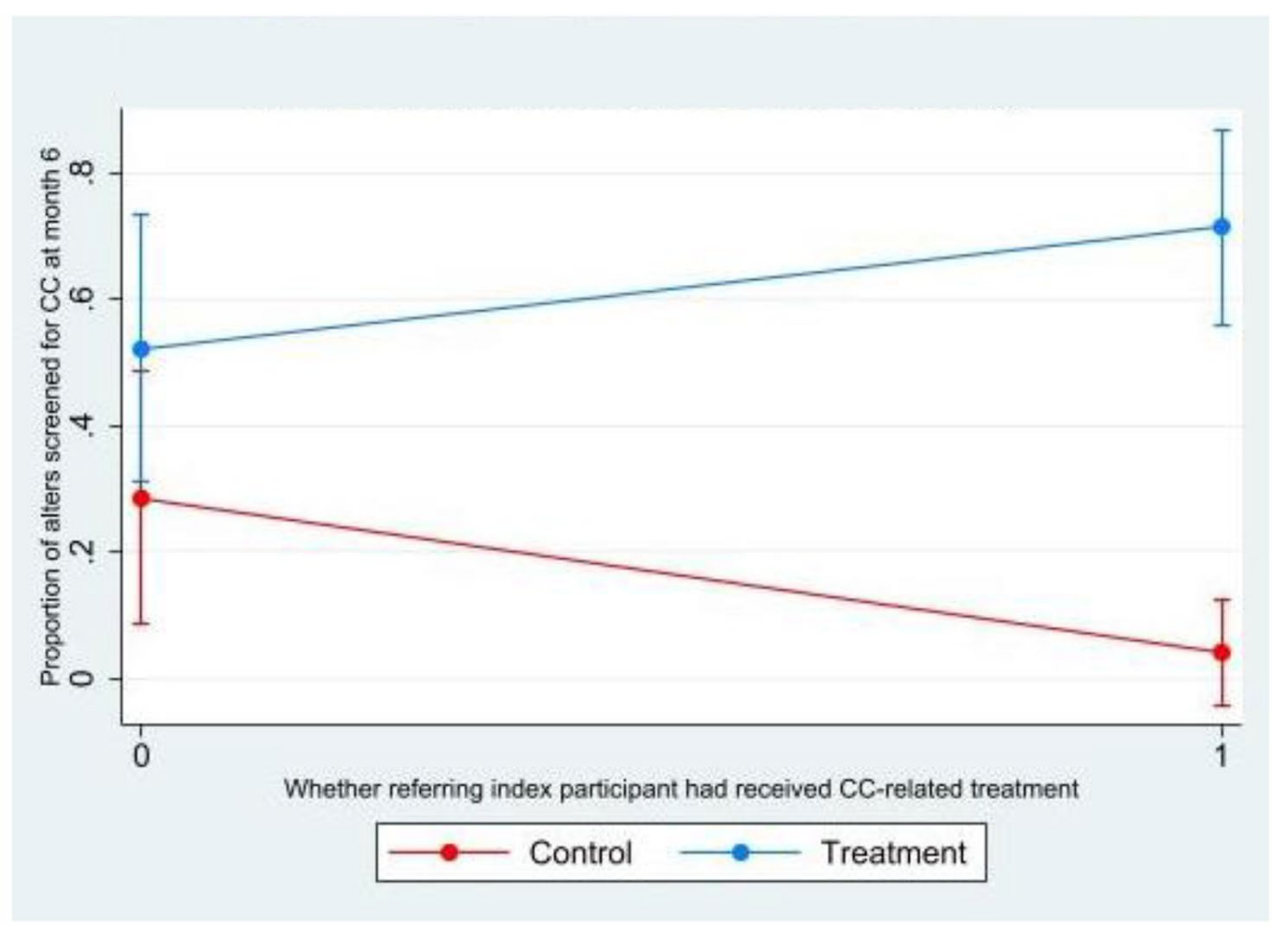
Moderating effects of index participant’s CC-related treatment status on the intervention effect on alter CC screening

In regression models examining moderation, receipt of thermal therapy for precancerous lesions by index participants was the only significant moderator of the intervention effect on alter CC screening [Adj. OR (95% CI) = 21.08 (1.78, 249.82)]. Figure 2 shows the scatterplot that depicts the interaction of the intervention with CC treatment status of the index participant who recruited the alter and its relationship to alter CC screening at month 6. The CC treatment status of the index participant interacted with the intervention such that alters in the intervention arm were more likely to have gotten screened for CC if their recruiting index had received thermal therapy, compared to alters in the control group who were less likely to get screened if their recruiting index participant had received CC-related treatment.

## Discussion

Prior analysis of the study data revealed an effect of the GC-CCP intervention on increased CC screening uptake among previously unscreened social network members (alters) (15). In the analysis reported here, we sought to understand how the intervention achieved this effect, and for whom the intervention was most successful, by examining mediators and moderators of the intervention effect on alter CC screening. The most direct effect of the intervention was engagement in CC prevention advocacy among intervention recipients (index participants); therefore, our hypothesis was that CC prevention advocacy conducted by the index participants would mediate the intervention effect on increased CC screening uptake among alters. Yet, while there was an intervention effect on increased CC prevention advocacy among index participants (Wagner et al. *in press*), it was increased engagement in CC screening advocacy among alter participants that fully mediated the intervention effect on alter uptake of CC screening. Also, the intervention effect was most prominent among alters who were recruited by index participants who had screened positive and received treatment for pre-cancerous lesions.

The GC-CCP intervention had effects on many of the constructs that compose the conceptual framework that guided the development of the intervention (see Fig. 1). Among index participants, the intervention led to significant increases in sharing of CC screening experience, CC knowledge, CC risk management self-efficacy, CC prevention advocacy self-efficacy, and engagement in CC prevention advocacy (as well as CC screening advocacy more specifically). However, none of these index measures of CC-related processes mediated the intervention effect on alter CC screening. The intervention also had effects on alter CC-related processes—namely, increased CC knowledge, CC risk management self-efficacy, and engagement in CC prevention advocacy—and it was the increased alter engagement in CC prevention advocacy that fully mediated the intervention effect on alter CC screening.

The mediating effect of increased alter engagement in CC prevention advocacy, as opposed to index engagement in advocacy, perhaps should not be surprising. The increase in alter engagement in advocacy is arguably a result of the alters being targeted with advocacy by the index participants. Furthermore, the finding that engagement in CC prevention advocacy also increased among alter participants suggests a diffusion of advocacy created by the intervention. This is particularly promising, as network-based peer advocacy interventions such as GC-CCP are designed to disseminate knowledge, motivation and self-efficacy for health behavior through diffusion of advocacy [10, 19, 20]. Future research will need to assess how far into the network (and away from direct exposure to the intervention) advocacy is diffused, and at what point the effects of advocacy on alter uptake of CC screening taper off.

The only variable found to moderate the intervention effect on alter CC screening was CC treatment status of the index participants. Index participants in the intervention group who had screened positive and been treated for pre-cancerous lesions were more likely to have alter participants who got screened for CC during the study follow-up period. This finding suggests that women who screen positive may be more motivated to engage in CC prevention advocacy, and they may also receive more information about CC during the process of receiving treatment, both of which encourage them to engage in more advocacy. This is consistent with our prior analysis of baseline data that showed index participant engagement in CC prevention advocacy was positively correlated with having received CC-related treatment (Wagner et al., *under review*). The advocacy of treated women may also be considered more credible by alters, given their experience of screening positive and receiving treatment. This finding does not discount the value of training women who screened negative to engage in advocacy, as many of these women in the intervention group also had alters who got screened for CC during the course of the study. Relatively few women screen positive, so training women who screen negative is important for achieving optimal community dissemination of CC knowledge and CC screening activation.

There are several limitations to our analysis. A selection bias was likely present in the recruitment of the index participants, as they had decided to enroll in a study that would train them to engage in CC prevention advocacy; motivation to be such an advocate is likely associated with greater CC knowledge and other constructs we measured, and not representative of the general population of women who had recently screened for CC. Similarly, there was a selection bias related to the alter participants, as index participants needed to be comfortable recruiting these alters; these alters may not be representative of all women in the social networks of the index participants. Validated measures or measures used by other research groups were not available for most constructs, resulting in the need for our team to develop many of the measures used. Internal reliability statistics were moderate or good for most of these measures, but further psychometric evaluation is needed in future studies. Other limitations include the small sample size and limited statistical power.

## Conclusion

Our study shows that the benefits of GC-CCP on alter uptake of CC screening are enhanced by the recipient of advocacy increasing her own advocacy for CC prevention and screening. The GC-CCP intervention had strong effects on nearly all components of the intervention, including index participants’ engagement in prevention advocacy. However, it was the alters’ engagement in prevention advocacy themselves that fully mediated the effect of the intervention on alter screening. The intervention effects on increased engagement in CC prevention advocacy and CC knowledge, among both index and alter participants, suggest a potential diffusion of advocacy and CC information throughout the network, which bodes well for CC screening activation throughout the network and the larger community.

## Data Availability

The datasets generated and/or analysed during the current study are available from the corresponding author on reasonable request.

## Abbreviations

CC: Cervical cancer
CI: Confidence Interval
GC-CCP: Game Changer Cervical Cancer Prevention [Intervention]
HPV: Human papillomavirus
OR: Odds Ratio
Adj. OR: Adjusted Odds Ratio
RHHJ: Rays of Hope Hospice Jinja
VIA: Visual inspection using acetic acid
WHO: World Health Organization
